# A thermo-reversible silicone elastomer with remotely controlled self-healing†

**DOI:** 10.1039/c7ra13686b

**Published:** 2018-02-22

**Authors:** E. Ogliani, L. Yu, I. Javakhishvili, A. L. Skov

**Affiliations:** Danish Polymer Centre, Department of Chemical and Biochemical Engineering, Technical University of Denmark Søltofts Plads, Building 229, 2800 Kgs Lyngby Denmark al@kt.dtu.dk

## Abstract

Soft thermoplastic elastomers with increased durability and reliability are in high demand for a broad spectrum of applications. Silicone elastomers are soft and durable, but they are not thermoplastic in nature, and under extreme conditions such as high voltage or large deformations, reliability may also suffer. Thus, as a solution to these shortcomings, which are typical of silicone elastomers, it is natural to propose a thermo-reversible, self-healing, and recyclable silicone-based elastomer. Stimuli-responsivity is imparted to the silicone polymer by incorporating supramolecular 2-ureido-4[1*H*]-pyrimidone (UPy) self-assembling motifs *via* free radical polymerisation. Self-healing of the novel elastomer may be triggered by both direct and indirect heating, the latter by means of incorporating Fe_3_O_4_ particles into the elastomer and subsequent exposure to an alternating magnetic field. As a consequence of temperature responsiveness and high thermal stability, the elastomer is proven recyclable, by withstanding multiple reprocessing procedures with no substantial effects on the resulting properties. The synergy of these valuable characteristics makes this novel material a smart candidate for innumerable applications where soft and reliable elastomers are sought.

## Abstract 2

A silicone elastomer with high healing efficiency due to supramolecular self-associating hydrogen bonding motifs has been synthesised.

## Graphical abstract

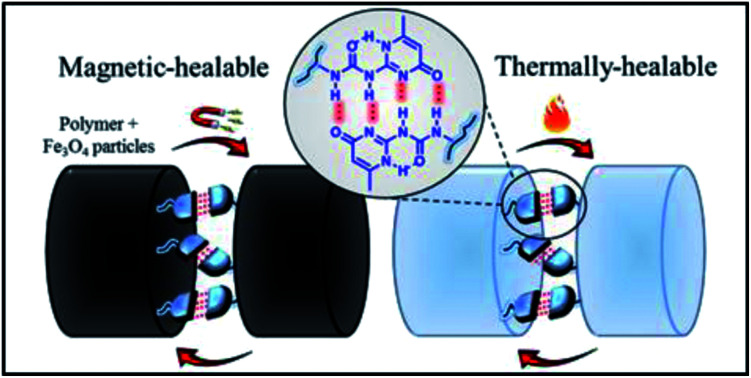

## Introduction

Polymeric materials with properties resembling those of biological systems, such as inherent flexibility, stretchability, and resilience to damage by self-healing, are in high demand. Self-healing materials are usually able to regain their properties fully or partially after damage or fracture. Thus, the life-time of a product based on the given material is significantly increased and its performance boosted. Different approaches have been conceived to achieve this desirable property, and the two main strategies are so-called “extrinsic” and “intrinsic” self-healing.^1^ The extrinsic approach consists of incorporating healing agents in capsules^2^ or micro-channels^3^ embedded in the polymer matrix. This usually leads to the possibility of one or limited^4^ cycles of self-healing only, since capsules are emptied after the self-healing process. In contrast, intrinsic self-healing is based on the inherent reversibility of the polymer matrix, and it can be triggered by diverse stimuli such as heat,^5^ light,^6^ and electricity.^7^ For this approach, the self-healing is not local, but will be reversible. Among the reversible non-covalent interactions (*e.g.* host–guest,^8^ hydrophobic,^9^ ionic interactions,^10^ π–π stacking interactions,^11^ and coordination bonding^12^), multiple hydrogen bond motifs offer remarkable potential for the design of supramolecular dynamic polymer networks since they possess high selectivity and directionality. In the design of reversible self-assembling polymer systems, 2-ureido-4[1*H*]-pyrimidone (UPy)^13^ is one of the most exploited associating groups, owing to its unique features of a self-complementary DDAA (donor–donor–acceptor–acceptor) array of four hydrogen bonds.^14,15^ In previous work, we reported the synthesis of 2-methoxyethyl acrylate (MEA) and 6-methyl-2-ureido-4[1*H*]-pyrimidone-bearing methacrylate (UPyMA) random copolymers,^16^ with the aim of clarifying the dynamics of hydrogen bonds in un-entangled associating polymers. Understanding the process of breaking and reforming these associations allows the manipulation of associative polymer properties, in order to tailor them to diverse applications.

Polydimethylsiloxane (PDMS) elastomers are utilised in an impressively wide range of applications, such as artificial muscles in the shape of dielectric elastomers where softness is sought,^17^ medical implants,^18^ soft robotics,^19^ microfluidic devices^20^ and many commodities (bakeware, cosmetics, and electronics amongst others). Despite their remarkable versatility and valuable properties, however, silicone elastomers are thermoset polymers due to their covalent nature, thus they are not easily recyclable. Therefore, it is a demanding challenge to overcome this drawback without sacrificing their more favourable properties. A few examples of thermoplastic elastomers (TPEs) have been reported. One method is the development of silicone-modified thermoplastic copolymers, such as polyurethane/silicone elastomers,^21^ or the introduction of physical cross-links through incorporating self-associating groups in the silicone backbone.^22^ The introduction of hydrogen bonds is a useful technique to provide silicone elastomers with not only thermoplastic properties, but also self-healing functionalities. Examples of supramolecular PDMS elastomers based on hydrogen bonding include the work of Yang *et al.*,^23^ who reported the synthesis of a silicone-based TPE through the reaction of carboxyl-terminated PDMS with diethylenetriamine (DETA) and urea, and Tazawa *et al.*,^24^ who designed thermoplastic PDMS with l-phenylalanine-based hydrogen bond networks. Nevertheless, the self-healing processes in the previous approaches require a long time or lead to a drastic decrease in mechanical properties.

Beyond the thermoplasticity of the material, self-healing properties have been sought by alternative approaches. Corten *et al.*^25^ introduced induction heating as a novel approach to generate heat and externally trigger polymer healing. This method consists of incorporating magnetic particles into the polymer matrix and the subsequent exposure of the composite to an alternating magnetic field (AMF). Consequently, the magnetic particles dissipate energy to the surrounding matrix, due to friction between the oscillating magnetic particles and the stationary matrix. The heat developed by the process allows for thermo-responsiveness and network rearrangement. AMF generates heat rapidly and locally, compared to supplying heat directly to the thermally insulating elastomer. Furthermore, self-healing is easily tunable by manipulating diverse parameters, such as frequency of the applied magnetic field, the nature of the susceptor material, and particle loading. Above all, the process is contactless. For instance, contactless activation becomes of interest when the material is employed as part of a component which is difficult to access, *e.g.* intermediate components, coatings and seals in the automotive industry.^26^

In this paper, we report the magnetic field-triggered self-healing of a novel thermoplastic silicone elastomer, namely P(PDMSMA-*co*-UPyMA), resulting from the free radical polymerisation of monomethacryloxypropyl terminated polydimethylsiloxane (PDMSMA) and UPyMA monomers. This novel copolymer possesses thermoplastic properties because of the reversible nature of UPy self-associating dimers. Self-healing of the material is activated *via* direct supply of heat as well as remotely by induction heating. Remotely controlled healing has been proven through exposure of the composite with 20 wt% Fe_3_O_4_ particle filler to an AMF. Moreover, the elastomer can be remoulded multiple times, without showing noteworthy chemical or physical degradation. Hence, this novel material may be considered an excellent candidate for recyclable silicone elastomers. In addition, the described self-healing approach, using the same conditions, has been applied to the previously reported material P(MEA-*co*-UPyMA).^16^ The P(PDMSMA-*co*-UPyMA) and P(MEA-*co*-UPyMA) copolymers bear identical self-complementary hydrogen-bonding motifs, albeit differing in the main repeating unit. Comparing their self-healing performance under identical conditions aims at proving how the optimised self-healing procedure is versatile and not system-dependent.

## Experimental

### Materials

*N*,*N*-Dimethylformamide (DMF; Sigma-Aldrich, ≥99.9%) was dried over molecular sieves. α,α′-Azoisobutyronitrile (AIBN; Ventron) was re-crystallised from methanol. Monomethacryloxypropyl terminated polydimethylsiloxane (PDMSMA MCR-M07; Gelest, 703.3 g mol^−1^ as determined by ^1^H NMR), 2-isocyanatoethyl methacrylate (Sigma-Aldrich, 98%), 2-amino-4-hydroxy-6-methylpyrimidine (Sigma-Aldrich, 98%), iron (ii, iii) oxide powder (Fe_3_O_4_; Sigma-Aldrich, size < 5 μm, 95%), tetrahydrofuran (THF; Sigma-Aldrich, 99.9%), methanol (Sigma-Aldrich, 99.9%), dimethyl sulfoxide (DMSO; SAFC, ≥99%), 1,4-dioxane (Sigma-Aldrich, 99.8%), and THF-*d*_8_ (Sigma-Aldrich, 99.8 atom% D) were used as received. 6-Methyl-2-ureido-4[1*H*]-pyrimidone-bearing methacrylate (UPyMA) was synthesised as reported in the literature.^15^

### Analytical techniques

Nuclear magnetic resonance (NMR) experiments were carried out on a Bruker Avance 300 MHz spectrometer. Attenuated total reflectance Fourier transform infrared (ATR FTIR) spectra in the range of 4000–350 cm^−1^ were recorded on a Nicolet iS50 ATR spectrometer with a diamond crystal from Thermo Scientific. To run size exclusion chromatography, a Viscotek 200 instrument was used, provided with two PLgel mixed-D columns (Polymer Laboratories (PL)) assembled in series, and a refractive index detector, utilising THF (1 mL min^−1^) as the mobile phase operating at room temperature. Molecular weights were calculated using polystyrene (PS) standards from PL employing TriSEC software. Scanning electron microscopy (SEM) and microanalysis were performed with an FEI Quanta 200E-SEM environmental scanning electron microscope, equipped with a field emission gun. The surface was visualised in a low vacuum, using water vapour as auxiliary gas at a pressure of 150 Pa. A mixture of secondary and back-scattered electrons, generated by the sample surface, was detected with the large field detector for an incident electron beam of spot 3 accelerated to 10 keV. The elemental composition of the samples was determined by energy dispersive X-rays (EDX) with an Oxford Instruments 80 mm^2^ X-Max silicon drift detector Mn Kα resolution at 124 eV, also in a low vacuum (150 Pa) with a 500 μm pressure-limiting aperture X-ray cone. Microanalysis data acquisition and quantification were performed with the Oxford Instruments Aztec program version 3.1.

### Synthesis of P(PDMSMA-*co*-UPyMA)

A Schlenk tube was charged with PDMSMA (0.97 mL, 0.43 mmol), AIBN (23 mg, 0.14 mmol), DMF (3.6 mL) and 1,4-dioxane (3.6 mL) along with comonomer UPyMA (302 mg, 1.08 mmol). The reaction mixture was stirred and deoxygenated by bubbling nitrogen through it for 45 min. The tube was then immersed in an oil bath at 90 °C, and polymerisation was carried out for 24 h. Thereafter, the tube was taken out of the bath and the reaction mixture exposed to the air. It was then precipitated twice from THF in a methanol-deionised water (3 : 1) mixture. The product was dried in the vacuum oven until no residual solvent was detected by spectroscopic means (yield > 60%).

### Preparation of the magnetic composite

P(PDMSMA-*co*-UPyMA) and P(MEA-*co*-UPyMA) magnetic composites were prepared by solvent casting a concentrated solution of the copolymers in THF and DMF, respectively. Subsequently, 20 wt% Fe_3_O_4_ particles (size less than 5 μm) were added to the concentrated solution (700 mg mL^−1^), which was placed in an ultrasonic bath for 30 minutes in order to ensure the homogeneous dispersion of particles inside the matrix. The solution was cast in a metal frame (4 × 4 × 0.1 cm^3^) and the sample was left at room temperature or in an oven at 40 °C, to allow for the slow evaporation of the solvent. The dried films were then removed from the frame and used for the experiments.

### Thermal properties

Thermal transitions were measured in the range −150 °C to 180 °C at a heating rate of 20 °C min^−1^, on a differential scanning calorimeter (DSC) Discovery series from TA Instruments. Thermogravimetric analysis (TGA) was performed from room temperature to 700 °C at a heating rate of 10 °C min^−1^ and an inert atmosphere on a thermogravimetric analyser Discovery series (TA Instruments).

### Linear viscoelastic measurements

Linear viscoelastic (LVE) properties of the polymer and its composites were measured with an ARES-G2 rheometer (TA Instruments), setting the instrument to a controlled strain mode, with 1% strain and frequency sweeps from 100 Hz to 0.01 Hz at 25, 50 and 70 °C using parallel-plate geometry 25 mm in diameter.

### Tensile properties

The tensile stress and tensile strain of the samples, with and without Fe_3_O_4_ particles and after self-healing, were measured with an ARES-G2 rheometer (TA Instruments) on a series of rectangular specimens (18 mm length, 10 mm width and ∼0.7 mm thickness) by uniaxial extensional rheology, using an ARES-G2 rheometer with an SER2 geometry. The test specimen was elongated uniaxially at a steady Hencky strain rate of 0.01 s^−1^ until sample failure. All measurements were repeated three times and results are reported as the mean value of tensile stress and tensile strain at breaking, with respective standard deviation.

### Alternating magnetic field-triggered self-healing

Self-healing experiments were performed using a MagneTherm instrument from Nanotherics, operating at a wide range of frequencies and consisting of a power supply, a function generator, an oscilloscope and a coil enclosure connected to a cooling system. Prior to the AMF tests, the frequency was set at 110.1 kHz (corresponding to a magnetic field strength of 250 Oe), the composite sample was placed in the centre of a 17-loop induction coil and the experiments were then performed. A non-contact IR gun thermometer was used to detect temperature changes in the sample during AMF measurements. AMF experiments were performed in cycles of 20 minutes to avoid overheating the induction coil.

### Synthesis of P(MEA-*co*-UPyMA)

P(MEA-*co*-UPyMA) was synthesised through a free radical polymerisation protocol as reported in the literature.^16^

## Results and discussion

### Characterisation of P(PDMSMA-*co*-UPyMA)

**Fig. 1:**
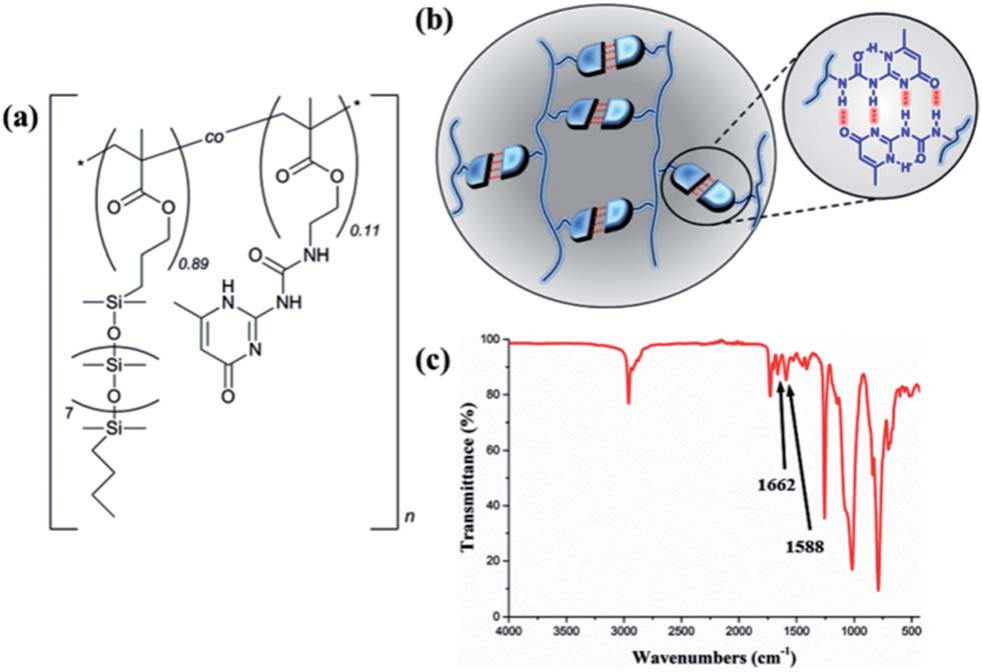
(a) Chemical structure of P(PDMSMA-*co*-UPyMA), (b) schematic representation of multiple hydrogen-bonding interactions, and (c) FTIR spectrum of P(PDMS-*co*-UPyMA).

The thermoplastic silicone-based copolymer was synthesised *via* free radical polymerisation of the PDMSMA and UPyMA monomers, resulting in a random distribution of the hydrogen bonding groups along the backbone. Fig. 1a depicts the structure of the copolymer, comprising pendant, thermo-reversible UPy dimers (Fig. 1b). Successful synthesis of the copolymer was confirmed using ^1^H NMR (Fig. S1†) and Fourier-transform infrared spectroscopy (FTIR, Fig. 1c). FTIR spectroscopy revealed characteristic absorption bands at 1662 and 1588 cm^−1^ belonging to the urea and pyrimidinone groups, respectively. The UPyMA molar fraction in the copolymer was quantified to 11% on the basis of ^1^H NMR analysis (Fig. S1†). The weight-average and number-average molecular weights, polydispersity index (PDI), and thermal properties are reported in ESI (Fig. S2 and S3†).

### Characterisation of the copolymer and the magnetic composite

**Fig. 2:**
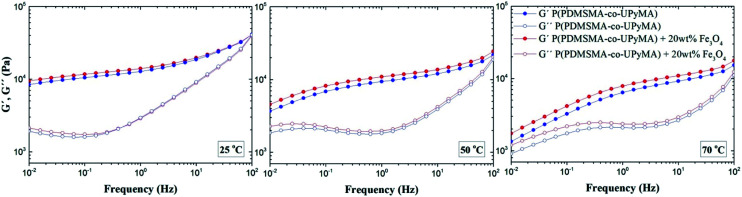
Small-amplitude oscillatory shear rheology: shear storage moduli *G*′ and shear loss moduli *G*′′ of pure P(PDMSMA-*co*-UPyMA) and the magnetic composite measured at 25 °C, 50 °C and 70 °C.

Small-amplitude oscillatory shear rheology was performed to investigate the dynamic nature of the copolymer network and the influence of the magnetic filler on the linear viscoelastic properties of the material. Fig. 2 illustrates storage (*G*′) and loss (*G*′′) moduli as a function of frequency at different copolymer temperatures, with and without Fe_3_O_4_ particles. The rheological spectra of pure P(PDMSMA-*co*-UPyMA) and magnetic composite are very similar and show viscoelastic behaviour with a dominant elastic plateau at fast deformation. When the test temperature was increased, the width of the elastic plateau decreased and the terminal flow behaviour became evident at low frequencies. In particular, the onset of the terminal flow is temperature-dependent and occurs at higher frequencies for increasing temperatures, as expected from time–temperature superposition principles. These results prove the dynamic nature and the thermo-reversibility of the bonds within the supramolecular network of the copolymer,^27^ which are fundamental to the self-healing properties. Moreover, it was observed that the presence of the filler did not affect significantly the viscoelastic properties of the copolymer, inducing an increase in the shear storage moduli at 0.01 Hz of 12% and 29%, at 25 °C and 70 °C, respectively.

To prepare the magnetic composite, the amount of magnetic filler was optimised in order to obtain the highest heat generation when exposed to an alternating magnetic field, without affecting the elastomeric nature of the material. The magnetic composite was then characterised by means of thermogravimetric analysis (TGA) and scanning electron microscopy (SEM). In particular, the actual value of the filler content in the composite material was assessed by TGA, which confirmed the expected value by the residual weight% detected at the end of the experiment (Fig. S4†). From SEM analysis, it was confirmed that the elastomer composites were homogeneous (Fig. S5†). A uniform distribution of the magnetic particles within the composite is a prerequisite for the even heating of the matrix, thereby avoiding the presence of cold and hot regions.^28^

### Evaluation of self-healing efficiency

The self-healing capability of the novel P(PDMSMA-*co*-UPyMA) was evaluated not only by directly heating the pure material in an oven, but also by indirectly heating the magnetic composite through exposure to an AMF. In both cases, the increase in temperature was used as a driving force to trigger the thermo-reversibility of the supramolecular self-associating UPy dimers. As stated by reptation theory,^29^ when a material is damaged, the healing process occurs at the cut interface through surface contact and rearrangement, wetting, diffusion, and randomisation. Hence, the main aim was to prove that this silicone-based elastomer could recover after damage as a result of the thermally activated rearrangements of the hydrogen bonds at the cut interface.

**Fig. 3:**
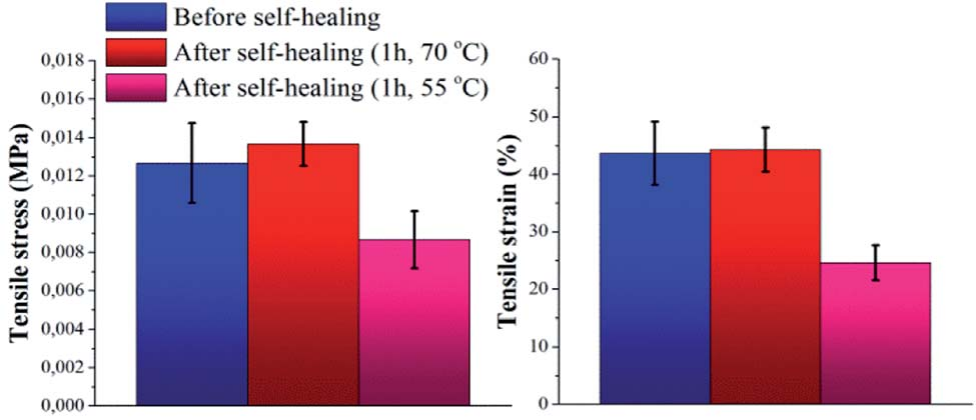
Tensile stress and tensile strain of pure P(PDMSMA-*co*-UPyMA), before and after healing for 1 h in oven at a pre-set temperature of 55 °C and 70 °C, respectively.

First, the self-healing ability of the pure copolymer was investigated by means of direct heating. Experiments were performed by cutting the specimen with a razor blade into two equal parts, which were then rejoined by contacting the cut surfaces. Subsequently, the damaged sample was placed in an oven. Self-healing properties were investigated after 1 hour at two different temperatures, namely 55 and 70 °C. At room temperature no self-healing was recorded after 1 hour. Tensile stress and tensile strain of the samples were measured before and after treatment in the oven, in order to evaluate the self-healing efficiency of the material (Fig. S6a and Table S2†). In particular, the self-healing efficiency was determined with respect to the percentage of restored tensile strain (*η*_*ε*_) and tensile stress (*η*_*σ*_).^30^Fig. 3 shows clearly how the mechanical properties of the material healed by direct heating at 70 °C after damage were fully restored, by comparison to the mechanical properties of the native copolymer. On the other hand, direct heating at a temperature of 55 °C only led to self-healing efficiencies corresponding to *η*_*ε*_ = 56% and *η*_*σ*_ = 66%. This behavior could be attributed to the limited amount of thermo-reversible UPy dimers present in the backbone (the UPy molar fraction in P(PDMSMA-*co*-UPyMA) was quantified to be 11%), so that an higher temperature is required to promote inter-diffusion of the chains through the interface and rearrangements of the hydrogen bonds. Nevertheless, the reported results indicate that the pure elastomer is capable of healing completely by means of small amounts of heat.

**Fig. 4:**
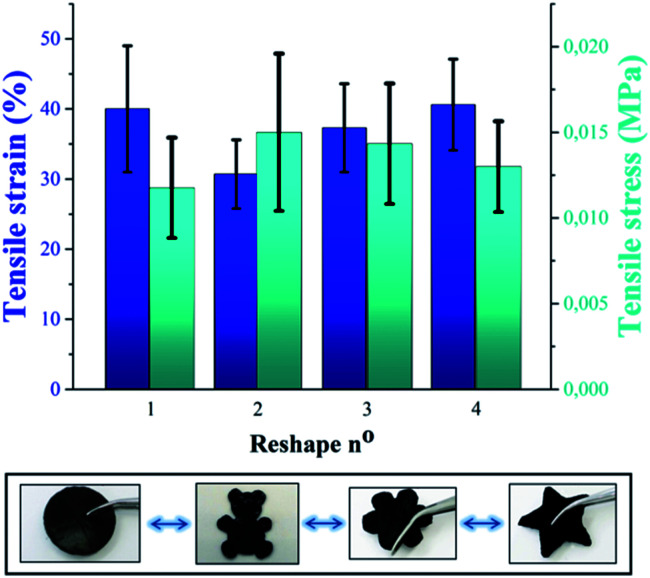
Comparison of tensile stress and tensile strain of P(PDMSMA-*co*-UPyMA) after repeated recycling by solvent casting. Multiple shapes obtained by consecutively reprocessing the material are displayed in the bottom figure (copolymer filled with iron oxide particles is used as an example).

Beyond self-healing capability, the recyclability of P(PDMSMA-*co*-UPyMA) was verified by means of solvent casting in tetrahydrofuran (THF). The tensile stress and tensile strain of the copolymer were measured after repeated reprocessing, and no significant changes in the values were observed (Fig. 4). Hence, the material can be reshaped and reused multiple times, without losing its original properties.

**Fig. 5:**
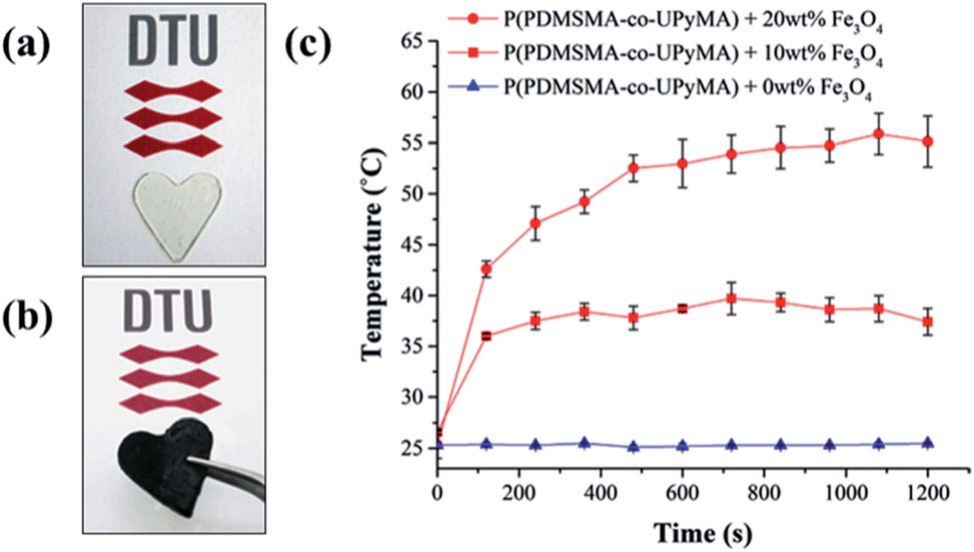
(a) Pure P(PDMSMA-*co*-UPyMA) and (b) P(PDMSMA-*co*-UPyMA) filled with iron oxide particles. (c) Heating temperature profiles of P(PDMSMA-*co*-UPyMA) filled with 0, 10, and 20 wt% particles and exposed to an alternating magnetic field at an applied frequency of 110.1 kHz.

Moreover, in this study, incorporating magnetic particles as a susceptor material inside the polymer matrix was employed to determine if the synthesised material is able to heal following exposure to an alternating magnetic field. Since the thermal response of the magnetic composite through exposure to an AMF depends on particle loading and the frequency of the magnetic field,^31^ these parameters were optimised to achieve the highest increase in temperature. Hence, a range of 10 frequencies was screened, in order to have a complete frequency–response profile of the magnetic particles embedded in the matrix. The best heating results were obtained at a frequency of 110.1 kHz, corresponding to a field strength of 250 Oe. Similarly, particle loading was optimised by evaluating the heating performance of the magnetic composite following exposure to AMF. As shown in Fig. 5, the composite with 20 wt% particle filler was found to have the highest final heating temperature of the bulk material, corresponding to 55 °C, and therefore it was chosen as a reference composite.

**Fig. 6:**
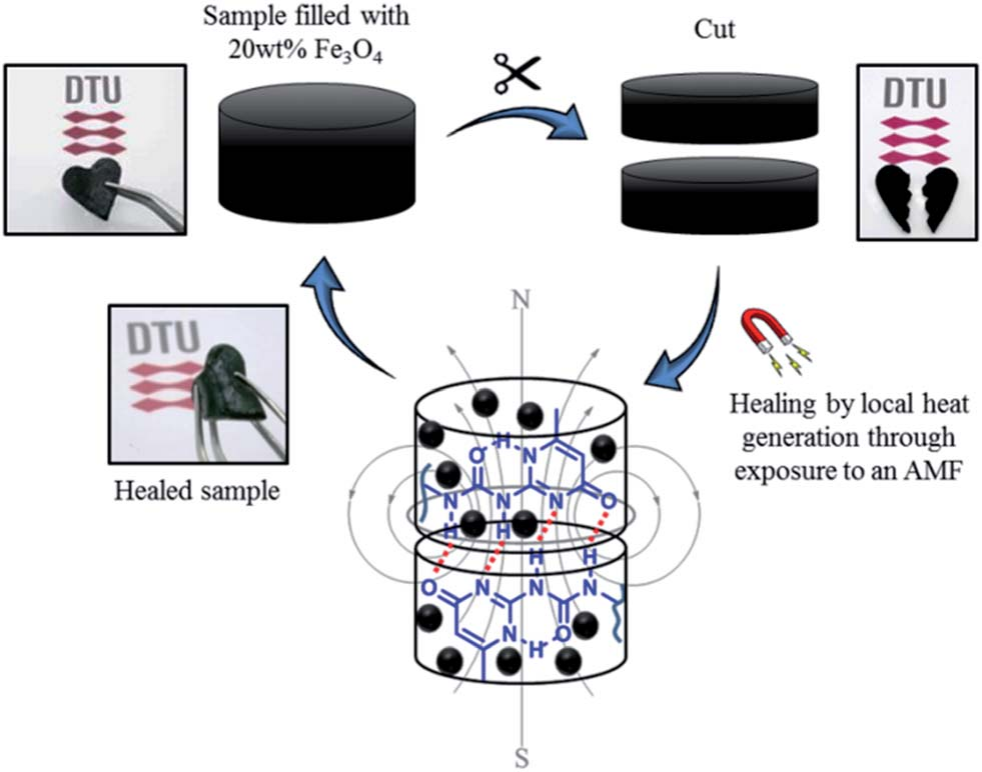
Schematic illustration of the alternating magnetic field mechanism activated self-healing of the novel P(PDMSMA-*co*-UPyMA) filled with 20 wt% iron oxide particles.

**Fig. 7:**
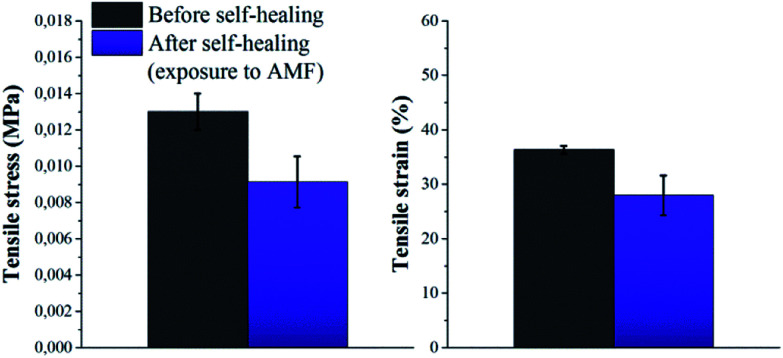
Tensile stress and tensile strain of P(PDMSMA-*co*-UPyMA) filled with 20 wt% iron oxide particles, before and after healing for 2 h (5 cycles) following exposure to an alternating magnetic field.

Based on these results, self-healing experiments were carried out by exposing magnetic composite with 20 wt% particle loading to an alternating magnetic field at a frequency of 110.1 kHz (Fig. 6). As described previously, the specimen was cut with a razor blade into two equal parts, which were subsequently rejoined. Then, the damaged sample was placed in the centre of the induction coil and an alternating magnetic field was applied. Fig. 7 compares the mechanical properties of the native composite and the damaged composite after exposure to AMF. Even though complete healing was not achieved, the composite exhibited promising self-healing efficiencies, calculated as *η*_*ε*_ = 78% and *η*_*σ*_ = 70% respectively. These values are higher than the ones reported for the self-healing of the pure material by direct heating at 55 °C. Even though the higher detected temperature upon exposure to AMF was 55 °C, it should be mentioned that the bulk temperature of the composite was probably higher than the detected one. The temperature was measured by an IR-gun thermometer, which in capable of detecting only the temperature on the surface of the sample.

**Fig. 8:**
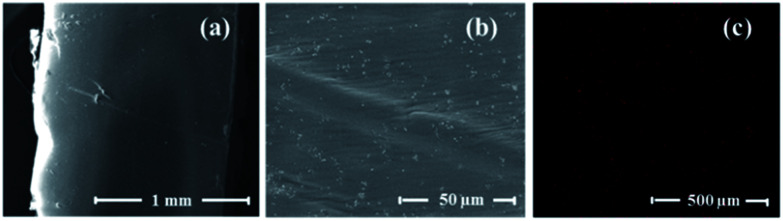
(a) and (b) SEM images of the magnetic composite filled with 20 wt% Fe_3_O_4_ after self-healing experiments following exposure to AMF and (c) elemental mapping of the specimen by EDS (iron distribution is visualised as red dots).

Moreover, scanning electron microscopy was exploited to observe the morphology of the cut interface of the composite after the self-healing experiments. In the SEM images in Fig. 8, the trace of the healed interface is discernible, and no gaps are visible. This suggests that the razor cut was filled with the material and, therefore, the polymer chains were able to diffuse through the damaged interface.^32^

After assessing the self-healing capability of the novel P(PDMSMA-*co*-UPyMA) by induction heating, the optimised procedure was subsequently exploited to investigate the self-healing performance of a previously reported copolymer synthesised by the free radical polymerisation of 2-methoxyethyl acrylate (MEA) and (UPyMA) monomers (Fig. S7†).^16^ P(MEA-*co*-UPyMA) and P(PDMSMA-*co*-UPyMA) are characterised by a similar UPy molar fractions, 6% and 11% respectively. Nevertheless, the difference in chemistry of the main repeating unit imparts significantly diverse characteristics to the materials. P(MEA-*co*-UPyMA) was filled with 20 wt% iron oxide particles, the specimen cut with a razor blade in the middle and then exposed to an AMF with the same applied frequency of 110.1 kHz and the same time of exposure (corresponding to five AMF cycles). Self-healing ability was evaluated by scanning electron microscopy (Fig. S8†). Similarly to P(PDMSMA-*co*-UPyMA), no gap was detectable in the cut interface, indicating that induction heating developed through exposure to AMF successfully promotes the rearrangement of hydrogen-bonding UPy groups. Even though self-healing efficiency should be quantified further, the results suggest that the self-healing method is versatile and may be effective in different systems.

## Conclusions

Two thermoplastic elastomers were developed based on supramolecular 2-ureido-4[1*H*]-pyrimidone (UPy) self-assembling motifs. Both elastomers possessed excellent self-healing properties and thermoplastic nature while maintaining their elasticity at room temperature (*G*′ approximately 10 fold larger than *G*′′). Furthermore both elastomers possessed fast self-healing times and were fully self-healed after 1 hour at 70 °C. The PDMSMA-*co*-UPyMA silicone elastomer proved to be remouldable multiple times with no detoriation of the properties and thus proving a robust and stable elastomer matrix for a thermoplastic silicone elastomer. Finally the silicone elastomer were loaded with magnetic particles to allow for self-healing by remote stimulus, namely by use of an alternating magnetic field. Efficient self-healing was also achieved by exposure to the alternating magnet, and the fillers were proven not to alter the elastomer properties to any significant extent.

## Conflicts of interest

There are no conflicts to declare.

## Supplementary Material

RA-008-C7RA13686B-s001
