# Supplementary material for: A thermo-reversible silicone elastomer with remotely controlled self-healing
Source: RSC Adv. 2018 Feb 22;8(15):8285–91. doi: 10.1039/c7ra13686b (PMC9078532; doi:10.1039/c7ra13686b)
Supplement: RA-008-C7RA13686B-s001 [file RA-008-C7RA13686B-s001.pdf]

Electronic Supplementary Material (ESI) for RSC Advances.
This journal is © The Royal Society of Chemistry 2018

Electronic Supplementary Material (ESI) for RSC Advances

Thermo-reversible silicone elastomer with remotely controlled self-healing

*Elisa Ogliani, Liyun Yu, Irakli Javakhishvili and Anne Ladegaard Skov**

Danish Polymer Centre, Department of Chemical and Biochemical Engineering, Technical University of Denmark, Søltofts Plads, Building 229, 2800 Kgs. Lyngby, Denmark.
E-mail: al@kt.dtu.dk

Table of Contents

Fig. S1. ^{1}H NMR of P(PDMSMA-co-UPyMA) in THF-d_8.

Fig. S2. Gel permeation chromatography of P(PDMSMA-co-UPyMA).

Fig. S3. Differential scanning calorimetry of P(PDMSMA-co-UPyMA).

Fig. S4. Thermogravimetric analysis of P(PDMSMA-co-UPyMA).

Fig. S5. SEM and EDS images of P(PDMSMA-co-UPyMA) filled with 20wt% Fe_3O_4.

Fig. S6. Stress-strain plot of pure P(PDMSMA-co-UPyMA) and P(PDMSMA-co-UPyMA) filled with 20wt% Fe_3O_4 before and after self-healing experiments.

Fig. S7. Chemical structure of P(MEA-co-UPyMA).

Fig. S8. SEM and EDS images of P(MEA-co-UPyMA) filled with 20wt% Fe_3O_4 before and after self-healing

Table S1. Number-average and weight-average molecular weights (M_n, M_w) and polydispersity index (PDI) of P(PDMSMA-co-UPyMA) obtained by GPC.

Table S2. Tensile stress, tensile strain, and self-healing efficiencies of P(PDMSMA-co-UPyMA) and P(PDMSMA-co-UPyMA) filled with 20wt% Fe_3O_4 before and after self-healing experiments.

Table S3. Molecular weight characteristics, composition and thermal properties of P(MEA-co-UPyMA).

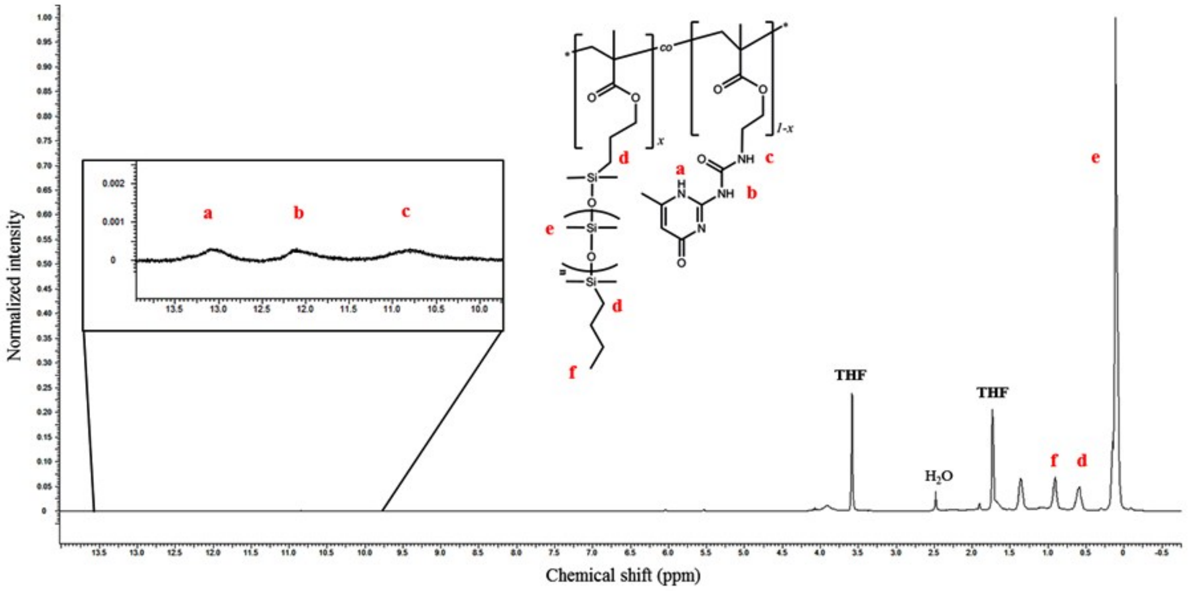

Fig. S1. ^{1}H NMR of P(PDMSMA-co-UPyMA) in THF-d_8.

The UPyMA molar fraction in P(PDMSMA-co-UPyMA) was quantified to be 11% on the basis of ^{1}H NMR analysis (Fig. S1). The calculation was made by comparison of the integrals of the resonance signals at 10.8, 12.2 and 13 ppm attributed to NH signals of the UPy group with the integral of the peak resonating at 0 ppm attributed to the protons of the methyl groups belonging to the -$Si(CH_3)_2O$- repeating units.

Gel permeation chromatography (GPC) was performed in order to determine weight-average and number-average molecular weights (M_w, M_n) and polydispersity index (PDI) of P(PDMSMA-co-UPyMA). GPC was carried out in tetrahydrofuran (THF) as the mobile phase. Fig. S2a shows the GPC traces. The presence of a peak below the minimum retention volume suggests that the strong hydrogen bonding interactions of the UPy dimers may induce the formation of high molecular weight aggregates. Therefore, small amounts of trifluoroacetic acid (TFA) were employed in order to break the hydrogen bonds and solubilize the copolymer before performing the GPC analysis. Fig. S2b and Table S1 summarize M_n, M_w and PDI obtained after the addition of TFA. A substantial decrease in the molecular weight is evident. In addition, after adding TFA, the peak at low retention volume was not observed in the GPC traces. These results may demonstrate that TFA could successfully facilitate dissociation of the self-associating UPy dimers. Moreover, Fourier-transform infrared spectroscopy (FTIR) was performed after treating the copolymer with TFA and subsequently drying the sample

(Fig. S2c). First of all, by comparing the spectra of the copolymer before and after treatment with TFA, it was found that the characteristic absorption bands at about 2959, 1256, 1017, and 791 cm^{-1}, which originate from the polydimethylsiloxane (PDMS) repeating unit were still detectable, suggesting that the addition of TFA did not lead to a considerable degradation of the PDMS pendant chains. Secondly, the band which appears at 3310 cm^{-1} after treatment with TFA may be assigned to the N-H stretch detectable only after dissociation of the hydrogen bonds. Thus, the addition of small amounts of TFA proved to be successful in order to break the high molecular weight aggregates, and perform size exclusion chromatography to obtain apparent molecular weights, however further analysis is required in order to determine the actual values of M_n and M_w of P(PDMSMA-co-UPyMA).

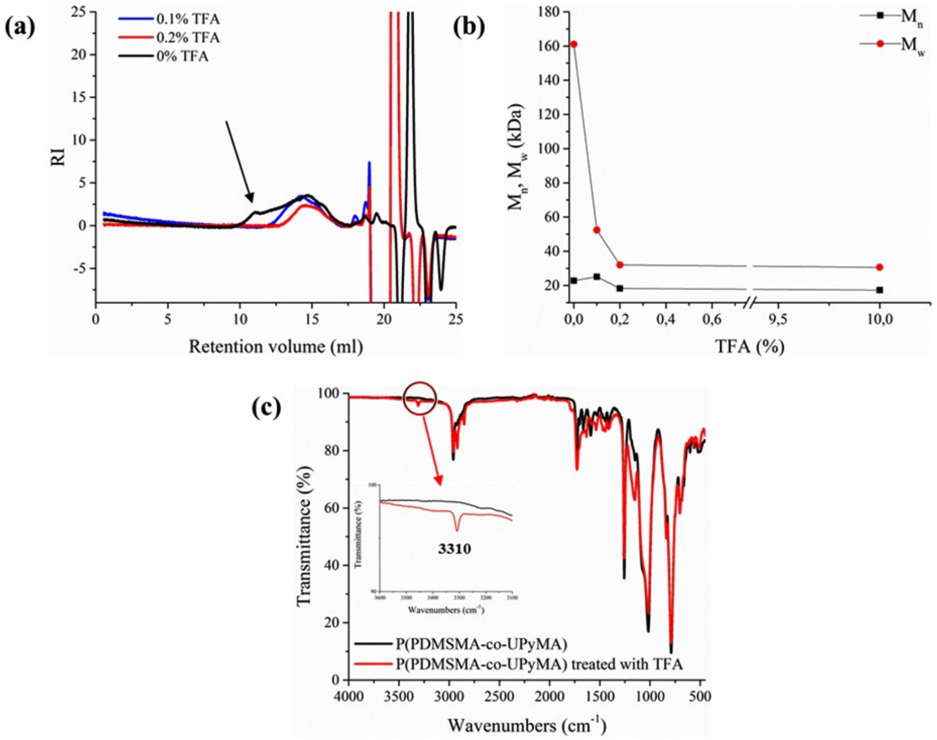

Fig. S2. a) GPC chromatograms obtained by treating P(PDMSMA-co-UPyMA) with different amounts of TFA. Percentage values are calculated from the total amount of TFA used to prepare the sample for GPC analysis. The black arrow points at the peak at low retention volume detected by GPC without the addition of TFA. b) Weight-average and number-average molecular weights obtained by GPC, and plotted as a function of the percentage of TFA. c) FTIR spectra of P(PDMSMA-co-

UPyMA) before and after treatment with TFA. The red arrow points at the absorption band at 3310 cm^{-1}, which appears after treatment with TFA.

Table S1. Number-average and weight-average molecular weights (M_n, M_w) and polydispersity index (PDI) of P(PDMSMA-co-UPyMA) obtained by GPC.

TFA (%)	0	0.1	0.2	10
M_n (kDa)	22.8	25.1	18.3	17.3
M_w (kDa)	161.1	52.4	32	30.6
PDI	7.1	2	1.7	1.8

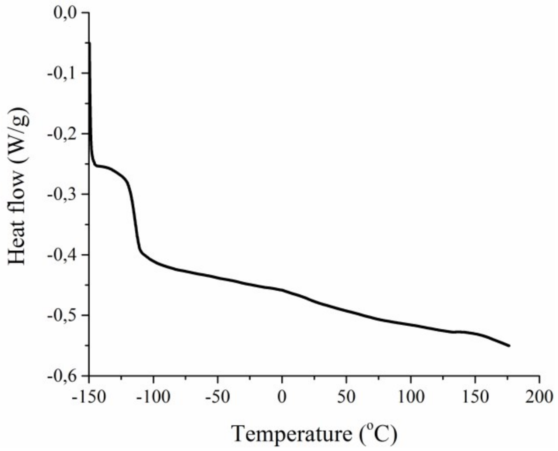

Fig. S3. Differential scanning calorimetry (DSC) curve of P(PDMS-co-UPyMA) at a heating rate of 20 ˚C min^{-1}.

The glass transition temperature (T_g) of P(PDMSMA-co-UPyMA) was measured by DSC (Figure S3), and it was found to correspond to -114 °C. The measurements was made by extrapolation of the midpoint of the endothermic stepwise change in the heat flow.

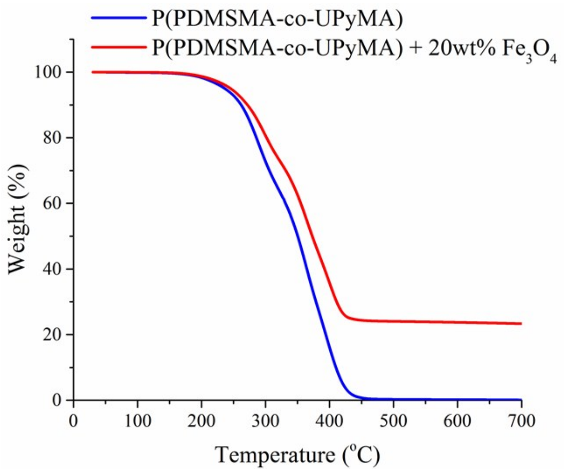

Fig. S4. Thermogravimetric analysis (TGA). Percentage of the weight loss plotted as a function of temperature of pure P(PDMSMA-co-UPyMA) and P(PDMSMA-co-UPyMA) filled with 20wt% Fe_3O_4 particles.

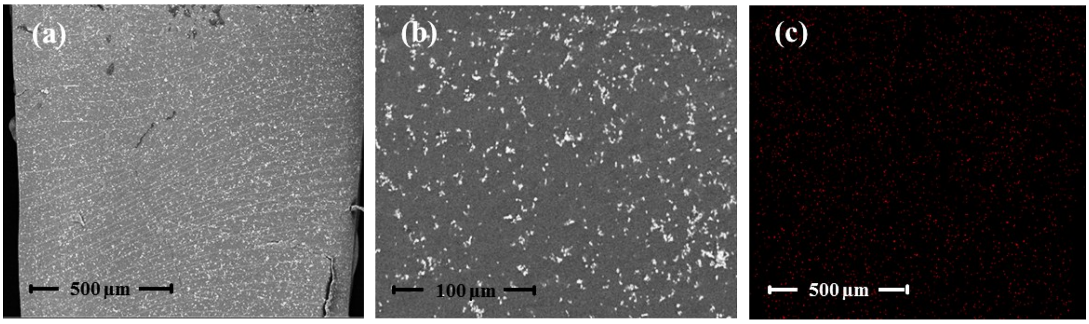

Fig. S5. a), b) SEM images of P(PDMSMA-co-UPyMA) filled with 20wt% Fe_3O_4 and c) elemental mapping of the specimen by EDS (iron distribution is visualized as red dots).

The distribution of magnetic particles within the P(PDMSMA-co-UPyMA) was investigated by scanning electron microscopy (SEM). Fig. S5a and S5b display SEM images of the cross section of the specimen recorded with a backscattered electron detector (BSED). Magnetic particles appear as white spots homogeneously dispersed within the surrounding matrix. In addition, elemental mapping of the specimen was performed in conjunction with SEM by energy-dispersive X-ray spectroscopy (EDX) technique. In Fig. S5c, EDS analysis shows concentration of iron (red dots) across the surface of the specimen and corroborates the homogeneity of magnetic particle distribution.

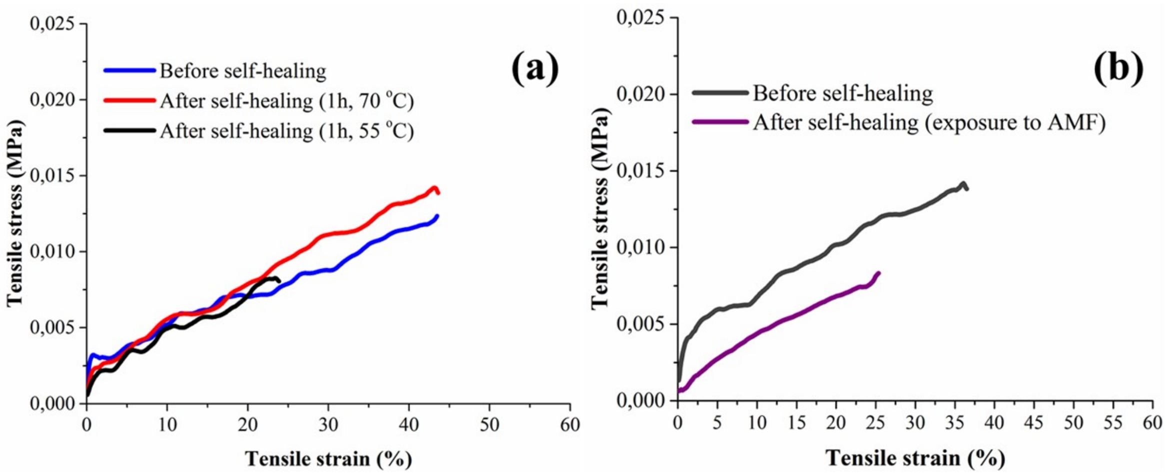

Fig. S6. a) Stress-strain plots of pure P(PDMSMA-co-UPyMA) before and after self-healing by direct heating and b) stress strain plots of P(PDMSMA-co-UPyMA) filled with 20wt% Fe_3O_4 particles before and after self-healing by exposure to an alternating magnetic field.

The Young´s moduli of the pure P(PDMSMA-co-UPyMA) and P(PDMSMA-co-UPyMA) filled with 20wt% Fe_3O_4 were calculated to be $2.8*10^{-4}$ MPa and $4*10^{-4}$ MPa.

Table S2. Tensile stress, tensile strain, and self-healing efficiencies of P(PDMSMA-co-UPyMA) and P(PDMSMA-co-UPyMA) filled with 20wt% Fe_3O_4 before and after self-healing experiments.

	Tensile stress [MPa]	Tensile strain [%]	Self-healing efficiency[a)] η_ε [%]	Self-healing efficiency[b)] η_σ [%]
P(PDMSMA-co-UPyMA)	0.012 ± 0,002	43.6 ± 5.5	--	--
P(PDMSMA-co-UPyMA) after self-healing (1h, 55˚C)	0.008 ± 0.0015	24.6 ± 3	56	66.6
P(PDMSMA-co-UPyMA) after self-healing (1h, 70˚C)	0.013 ± 0.0011	44.3 ± 3.8	100	100
P(PDMSMA-co-UPyMA) + 20wt% Fe_3O_4	0.013 ± 0.001	36 ± 0.7	--	--
P(PDMSMA-co-UPyMA) + 20wt% Fe_3O_4 after self-healing by exposure to AMF	0.0091 ± 0.0014	28 ± 3.6	77.7	70

[a)]Percentage of restored tensile strain; [b)]Percentage of restored tensile stress

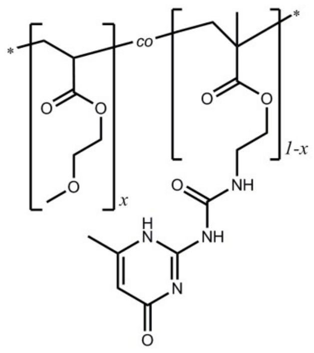

Fig. S7. Chemical structure of P(MEA-co-UPyMA).

Table S3. Molecular weight characteristics, composition and thermal properties of P(MEA-co-UPyMA).

	M_w [kDa][a)]	M_n [kDa][a)]	PDI[a)]	F_{UPyMA} [mol %][b)]	T_g [˚C][c)]
P(MEA-co-UPyMA)	22.9	10.5	2.2	6	-30

[a)]Obtained by GPC; [b)]Estimated by ^{1}H NMR; [c)]Obtained by DSC

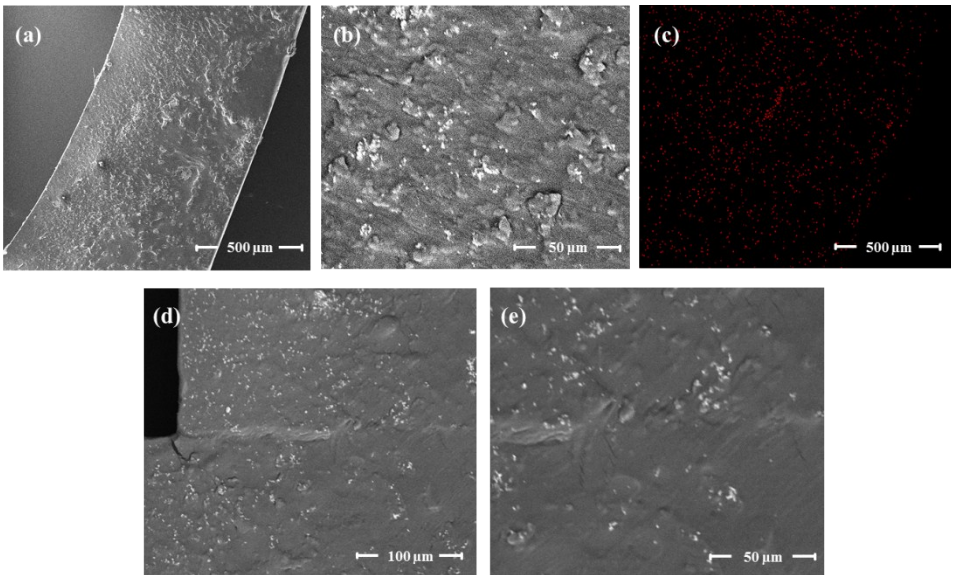

Fig. S8. a), b) SEM images of P(MEA-co-UPyMA) filled with 20wt% Fe_3O_4 and c) elemental mapping of the specimen by EDS (iron distribution is visualized as red dots). d), e) SEM pictures of P(MEA-co-UPyMA) filled with 20wt% Fe_3O_4 after self-healing experiments under exposure to an alternating magnetic field (AMF).

The distribution of magnetic particles within the P(MEA-co-UPyMA) was investigated by scanning electron microscopy (SEM). Fig. S8a and S8b display SEM images of the cross section of the

specimen recorded with an Everhart-Thornley detector (ETD) and a backscattered electron detector (BSED) respectively. Fe_3O_4 particles are shown as white spots dispersed within the surrounding matrix. Dispersion of magnetic particles is mainly proven homogeneous, even though the presence of small aggregates is detectable in the pictures. In Fig. S8c, EDS analysis shows concentration of iron (red dots) across the surface of the specimen and corroborates the homogeneity of magnetic particle distribution.
